# 

**Published:** 1914-01-03

**Authors:** 


### OLDEST MEDICAL OFFICER RESIGNS.

By the resignation of Dr. T. F. Higgs from the post of Medical Officer of Dudley Workhouse, perhaps the oldest medical officer ceases from active work. Dr. Higgs, who has filled the above position for fifty-two years, and who is over eighty years of age, is an M.D., L.R.C.P. (Edin.), M.R.C.S. (Eng.), senior hon. surgeon to the Dudley Dispensary, surgeon to the Dudley and Borough Police, and a Justice of the Peace.

### MEDICAL OFFICER FOR FORTY YEARS.

Dr. Henry J. Alford, Medical Officer of the Taunton Rural District Council, has tendered his resignation to that body after over forty years' service. The resignation is on account of the increase of duties occasioned in connection with the children of the elementary schools under the Borough Education Committee.

### Appointments.

Mr. W. E. Fox has been appointed fifth assistant medical officer at Bexley Mental Hospital.

Mr. Patrick Murphy has been appointed fifth assistant medical officer at Hanwell Mental Hospital.

Dr. G. W. FitzGerald has been appointed honorary surgeon for women at the Manchester Northern Hospital for Women and Children in place of Mr. Harold Clifford, resigned.

The vacancy on the surgical staff of the London Homœopathic Hospital has been filled by the election of Mr. James Johnstone, B.A., F.R.C.S. Eng., M.B., C.M., D.P.H., to be surgeon.

The following appointments have been made at Ancoats Hospital, Manchester:—Mr. F. S. Bedale, M.A., B.C. (Cantab.), M.R.C.S., L.R.C.P., house surgeon; Mr. F. B. Smith, M.A., B.C. (Cantab.), M.R.C.S., L.R.C.P., house physician; and Mr. F. S. Charnock, M.B., M.R.C.S., L.R.C.P., assistant house surgeon.

The following appointments and reappointments have been made at the Manchester Royal Infirmary:—Appointment: Mr. M. W. Paterson, B.A. (Cantab.), M.R.C.S., as medical officer at the Central Branch. Reappointments: Mr. A. E. Barclay, M.A., B.C. (Cantab.), M.D., M.R.C.S., L.R.C.P., as medical officer of the radiography and electricity department; Mr. S. P. Wilson, M.B., B.S. (Lond.), F.R.C.S. (Edin.), as second anæsthetist; Mr. T. Coogan, M.B., Ch.B. (Vict.), as fifth anæsthetist; Mr. G. E. Loveday, M.[illegible] B.C. (Cantab.), M.R.C.S., L.R.C.P., as director of the clinical laboratory; Mr. J. F. Ward, M.B., Ch.B. (Vict.), as resident medical officer of the Royal Infirmary.

Mrs. John Newton Mappin has been elected a vice-president of the Royal Sea Bathing Hospital for Surgical Tuberculosis, Margate.

RATES OF SUBSCRIPTION: Twelvemonths, 6/6; Six months, 4/-; Three months, 2/-, post free to any address in the United Kingdom. Abroad, Twelvemonths, 8/8; Six months, 5/-; Three months, 2/6, post free.—Address The Subscription Dept., "THE HOSPITAL," The Hospital Building, 28/29 Southampton Street, Strand, London, W.C.

